# An Interaction-Based Method for Refining Results From Gene Set Enrichment Analysis

**DOI:** 10.3389/fgene.2022.890672

**Published:** 2022-05-30

**Authors:** Yishen Wang, Yiwen Hong, Shudi Mao, Yukang Jiang, Yamei Cui, Jianying Pan, Yan Luo

**Affiliations:** ^1^ State Key Laboratory of Ophthalmology, Zhongshan Ophthalmic Center, Sun Yat-Sen University, Guangzhou, China; ^2^ Department of Statistical Science, School of Mathematics, Sun Yat-Sen University, Guangzhou, China

**Keywords:** GSEA, RNA-Seq, MiR-124-3p, apriori algorithm, miRNA

## Abstract

**Purpose:** To demonstrate an interaction-based method for the refinement of Gene Set Enrichment Analysis (GSEA) results.

**Method:** Intravitreal injection of miR-124-3p antagomir was used to knockdown the expression of miR-124-3p in mouse retina at postnatal day 3 (P3). Whole retinal RNA was extracted for mRNA transcriptome sequencing at P9. After preprocessing the dataset, GSEA was performed, and the leading-edge subsets were obtained. The Apriori algorithm was used to identify the frequent genes or gene sets from the union of the leading-edge subsets. A new statistic d was introduced to evaluate the frequent genes or gene sets. Reverse transcription quantitative PCR (RT-qPCR) was performed to validate the expression trend of candidate genes after the knockdown of miR-124-3p.

**Results:** A total of 115,140 assembled transcript sequences were obtained from the clean data. With GSEA, the NOD-like receptor signaling pathway, C-type-like lectin receptor signaling pathway, phagosome, necroptosis, JAK-STAT signaling pathway, Toll-like receptor signaling pathway, leukocyte transendothelial migration, chemokine signaling pathway, NF-kappa B signaling pathway and RIG-I-like signaling pathway were identified as the top 10 enriched pathways, and their leading-edge subsets were obtained. After being refined by the Apriori algorithm and sorted by the value of the modulus of d **,** Prkcd, Irf9, Stat3, Cxcl12, Stat1, Stat2, Isg15, Eif2ak2, Il6st, Pdgfra, Socs4 and Csf2ra had the significant number of interactions and the greatest value of d to downstream genes among all frequent transactions. Results of RT-qPCR validation for the expression of candidate genes after the knockdown of miR-124-3p showed a similar trend to the RNA-Seq results.

**Conclusion:** This study indicated that using the Apriori algorithm and defining the statistic d was a novel way to refine the GSEA results. We hope to convey the intricacies from the computational results to the low-throughput experiments, and to plan experimental investigations specifically.

## 1 Introduction

MicroRNAs (miRNAs) are a class of noncoding RNAs that play key roles in regulating gene expression and are involved in a variety of biological processes during retinal development (Damiani et al., 2008). In most cases, miRNAs inhibit the expression or promote the degradation of messenger RNA (mRNA) by interacting with the specific sequences located in the 3 UTR of their target mRNA (Ha and Kim, 2014). According to this feature, each miRNA can target hundreds of mRNAs. The development of transcriptomics technologies such as RNA sequencing (RNA-Seq) can provide a broad account of RNA transcripts and consequently insight into changes in the mRNA expression of downstream genes after experimental intervention on the miRNA (Wang et al., 2009).

Due to the large amount of sequencing data generated by RNA-Seq, appropriate bioinformatics methods are needed to handle the data. After preprocessing the data, traditional quantitative analysis for mRNA expression analysis focuses on identifying differentially expressed upregulated and downregulated genes between two individual groups. Traditional strategies usually set an arbitrary cutoff in terms of expression fold-change (e.g., fold-change ≥ 1.5 considered significant) to filter the critical genes. Conservative and relaxed cutoff values may cause false negative and false positive results, respectively, making the results less objective and reproducible. To overcome the analytical challenges of focusing on a single gene, gene set enrichment analysis (GSEA) helps to gain further insight into the distribution of genes preannotated in biological categories by incorporating the entirety of gene expression. However, both single and global gene analysis often generate a large number of candidate genes (Ackermann and Strimmer, 2009). The lack of golden standard datasets also makes the assessment of gene set analysis methods rudimentary (Maleki et al., 2020). Hence, instead of extracting more genes from datasets, the goal of this study was to attempt reducing the dimensionality of analysis results and refining the intricacies from the high-throughput results to the low-throughput experiments.

Several studies have characterized miRNA expression in the developing mammalian retina, and miR-124-3p has been shown to be one of the most abundantly expressed miRNAs (Karali et al., 2007; Hackler et al., 2010; Karali et al., 2010; Karali et al., 2016). Although the miR-124a-3p knockdown mouse exhibited neuronal dysfunction and dysmaturation by disinhibition of Lhx2, its downstream responses in mouse retinal development after birth were still relatively deficient at the transcriptome level. In our previous study, miR-124-3p exhibited a significantly increasing trend after birth, and its related pathways predicted by bioinformatics analysis were associated with biological processes that may play crucial roles in mouse retinal development (Wang et al., 2020). To gain insight into its downstream processes, RNA-Seq was used to obtain the expression profile of mRNA transcripts after the knockdown of miR-124-3p.

In GSEA, given a set of genes sorted depending on given conditions (e.g., mRNA expression level of downstream genes) and a biological category, a running sum statistic is computed iteratively from top to bottom of the sorted set to evaluate whether it is enriched in the given biological category. When processed, the running sum will increase whenever a gene belonging to the given biological category is found and otherwise decrease. Therefore, the running sum will be relatively high if the gene set falls at either the top (overexpressed) or bottom (underexpressed) and is likely to be subsequently related to the given biological category.

Based on the properties mentioned above, GSEA is an appropriate tool to obtain a precise description of the downstream effects of miRNAs. Since a miRNA and its target mRNAs demonstrate negative correlations because of degradation (Ritchie et al., 2009; Wang and Li, 2009), when the expression value of the miRNA is manipulated to decrease, the upregulated and downregulated mRNA expression can be assumed to be its direct effects and indirect effects, which are likely to be enriched at the top and bottom in GSEA, respectively, and vice versa.

Through the analysis of a downstream mRNA dataset from RNA-Seq after miR-124-3p knockdown, we demonstrate how the original GSEA method was extended and the results from GSEA were refined, which could be used as a comprehensive protocol for downstream analysis in the loss- and gain-intervention to a specific miRNA. Some parameters, such as the leading-edge subset, were modified to better describe the characteristics of the bottom (underexpressed) mRNAs based on the classical GSEA approach by Subramanian et al. (2005). The union of the leading-edge subsets in the enriched KEGG pathways was selected. For traditional GSEA, the number of generated candidate genes is usually still too high. One or several key genes or pathways need to be identified for further functional experiments. Apriori algorithm and a new statistic d were introduced to correct this issue. It is hypothesized that genes play critical roles in the entire leading-edge subsets if they have the most interactions with other genes. Apriori algorithm could use prior Boolean association rules (gene-gene interaction) to mine these pivotal genes or gene sets. Afterward, the expression vector of candidate genes or gene sets were modified with their relationship as a new statistic d . Finally, the candidate genes were refined, and key genes were identified.

## 2 Methods

### 2.1 Knockdown of miR-124-3p and RNA Extraction

C57BL/6J mice were used to study the transcriptome of miR-124-3p during retinal development. Mice at postnatal day 3 (P3) were given 1 μl of 0.6 nmol/μl miR-124-3p antagomir in the left eye as the anti-miR-124 group and 0.6 nmol/μl antagomir negative control in the right eye as the negative control (NC) group by intravitreal injection. Retinas from mice at P9 were harvested, and total RNA was isolated by TRIzol (Invitrogen; Thermo Fisher Scientific, Inc, Waltham, MA, United States) according to the manufacturer’s instructions. Both groups consisted of 12–15 mixed retina tissues, and one biological replicate was conducted. In addition, to increase the heterogeneity of the sample, the samples in each group were from at least two different litters.

### 2.2. RT–qPCR Validation for the Knockdown of miR-124-3p and RNA-Seq

Reverse transcription quantitative PCR (RT–qPCR) was performed to validate the knockdown rate of the anti-miR-124 group compared with the NC group. Total RNA from both groups was reverse transcribed using a PrimeScript RT reagent kit (Takara Bio, Inc, Otsu, Japan). Real-time PCR was subsequently performed on the resulting cDNA template with a TB Green™ Premix Ex Taq™ II kit (Takara Bio, Inc, Otsu, Japan) on a StepOnePlus™ Real-Time PCR System (Applied Biosystems; Thermo Fisher Scientific, Inc, Waltham, MA, United States). The 2^−ΔΔCt^ method was used to quantify miRNA expression levels with the u6 gene as an internal reference (Livak and Schmittgen, 2001). After significant knockdown of the expression level of miR-124-3p was confirmed by RT–qPCR, RNA-Seq was carried out to detect the expression levels of mRNAs in both groups. The RNA-Seq data in the present study are deposited in the Gene Expression Omnibus (GEO) repository, accession number GSE200915 (https://www.ncbi.nlm.nih.gov/geo/query/acc.cgi?acc=GSE200915).

### 2.3 GSEA

GSEA was carried out using Python 3.6 (Python Software Foundation. Python Language Reference, version 3.6, available at https://www.python.org).

#### 2.3.1 Data Preprocessing

To normalize the length of the mRNA sequences and the sequencing depth of a sample, transcripts per million (TPM) were used to assess the expression level of mRNAs. Read counts for all the mRNA transcripts were demonstrated in Data Sheet 1. The method for calculating TPM was described in detail elsewhere (Wagner et al., 2012; Dillies et al., 2013). Based on previous research, the frequency distribution of genes with different expression levels has a mode at TPM nearly equal to 0 and a long tail toward higher TPM values (Wagner et al., 2013; Bush et al., 2018; Monaco et al., 2019). Therefore, to determine an appropriate interval to filter genes with very low expression, a base 10 logarithmic scale was used to evaluate the frequency distribution of TPM for the genes in all groups.

#### 2.3.2 Calculating the Expression Difference

In each group set, the expression difference for a gene between the two groups was defined as the log2-transformed fold-change value of TPM. To reduce the false positive rate of the results, only genes that had the same expression trend between the two biological replicates were considered reliable. Based on these methods, a list of candidate genes and their corresponding gene expression differences was obtained.

#### 2.3.3 Obtaining the Enrichment Score and Its Related Parameters in Kyoto Encyclopedia of Genes and Genomes Pathways

The candidate genes were ranked from highest to lowest expression level, and the enrichment score (ES) for each Kyoto Encyclopedia of Genes and Genomes (KEGG) pathway was calculated based on the GSEA approach (Subramanian et al., 2005). KEGG gene annotation was derived from the KEGG database (https://www.genome.jp/kegg-bin/download_htext?htext=ko00001.keg&format=json&filedir=). After excluding three types of annotations (“09160 Human Diseases”, “09180 Brite Hierarchies”, “09,190 Not Included in Pathway or Brite”) in KEGG pathways that were not related to retinal development, GSEA was performed on 354 pathways whose expression patterns might have changed in the retina. Afterward, the *p* value for each KEGG pathway was estimated by comparing the absolute value of its maximum ES with randomly generated sets of absolute values of maximum ES. A *p* value <0.05 was considered significant or enriched. To balance the accuracy of the estimation and the required computing power, the number of permutations used to generate the comparisons was set to 1,000. For an enriched pathway, a leading-edge subset is a set of genes that contributes to the maximum ES in the ranked list of genes. The subset will be found at the top if the maximum ES is positive (upregulated gene subset) or at the bottom if the maximum ES is negative (downregulated gene subset). Afterward, the union of the leading-edge subsets in the enriched KEGG pathways was selected.

##### 2.3.3.1 Calculating the Maximum ES

Since a total of n candidate genes were ranked from highest to lowest expression level, we denoted $\delta =\left(\delta_{1},\delta_{2}\ldots \delta_{n}\right)$ as the vector of differences, where each element $\delta_{i}$ in δ represented the expression difference of a single gene $g_{i}$ between two groups. Accordingly, a corresponding gene vector $g=\left(g_{1},g_{2}\ldots g_{n}\right)$ was defined, where each element $g_{i}$ was the gene name using NCBI Gene Symbol. To calculate the ES in a KEGG pathway, it is necessary to determine whether the gene $g_{i}$ belongs to this pathway. We established the following definition: if $g_{i}$ belonged to the annotations of this pathway, $r\left(g_{i}\right)=1$ ; if $g_{i}$ did not belong to the annotations of this pathway, $r\left(g_{i}\right)=0$ , and a new vector $r=\left(r_{1},r_{2},\ldots r_{n}\right)$ was generated, where each element $r_{i}$ consisted of 0 and 1. To facilitate the iteration in the computer program, the calculation of ES was rewritten into the following form according to the method described by Subramanian *et al* (Subramanian et al., 2005).

$$es_{0}=0$$

$$es_{i}=r_{i}\frac{{\left|\delta_{i}\right|}^{p}}{N_{R}}+\left(r_{i}-1\right)\frac{1}{n-N_{H}}+es_{i-1},i\geq 1$$

where $N_{H}=\sum r_{i}$ , $N_{R}=\sum r_{i}{\left|\delta_{i}\right|}^{p}$ , $es_{i}$ represents the value of ES and p is a weighing factor with a range of [0, 1] . A value of p=1 was set as a constant in the study. By the method of mathematical induction, it was easy to prove that $es_{n}=0$ .

In particular, $N_{R}$ equal to 0 indicated that none of the genes in g belonged to the annotations in the KEGG pathway. To prevent a division by zero error (when $N_{R}=0$ ), we set any value of $es_{i}$ equal to 0 under this condition. Thus, the maximum ES, $es_{max}=sign\left(es_{k}\right)max\left(|es_{1}\left|,\;|es_{2}\right|\ldots |es_{n}|\right)$ , was obtained, where $\left|es_{k}\right|$ was the maximum element in the max(⋅) function, and sign(⋅) represented the signum function to indicate the positive and negative values of $es_{max}$ .

##### 2.3.3.2 Estimating the Significance of the Maximum ES

To estimate the significance of the $es_{max}$ generated from g in the KEGG pathway, a nominal *p* value was calculated using an empirical phenotype-based permutation method derived from Subramanian et al. (2005). Given the null hypothesis that the order of the elements in g was random, the way to reject the null hypothesis was to calculate the probability of $es_{max}$ in its randomly generated distribution, which was derived from a set of randomly assigned g . When the order of elements in g was disrupted and the other parameters remained unchanged, the new corresponding value of $es_{max}$ was related only to the random rearrangement of elements in g . After repeating the above random rearrangement process v times, a series of values of $es_{max}^{\prime }$ with a total number of v were obtained based on the null hypothesis, and the absolute value was taken: ( $\left|es_{max1}^{\prime }|,|es_{max2}^{\prime }|,\ldots |es_{maxv}^{\prime }|\right)$ . The empirical cumulative distribution function (CDF) ${\hat{F}}_{v}\;$ for $es_{max}^{\prime }$ (derived from the null hypothesis) can be described as follows:

$$\hat{F_{v}}\left(x\right)=\frac{1}{v}\overset{v}{\underset{i=1}{\sum }}I\left(|es_{maxi}^{\prime }|\leq x\right)$$

where I(⋅) is the indicator function of an event and v corresponds to the number of permutations in Subramanian *et al* (Subramanian et al., 2005). According to the empirical CDF, the *p* value for an $es_{max}$ in a pathway was calculated as follows:

$$p=1-\hat{F_{v}}\left(\left|es_{max}\right|\right)$$

##### 2.3.3.3 Obtaining the Leading-Edge Subsets

In an enriched pathway, the leading-edge subset in g appears prior to the peak score for a positive $es_{max}$ and appears subsequent to the peak score for a negative $es_{max}$ . The leading-edge subset L can be described as follows:

$$L=\{\begin{array}{c} \left\{g_{j}\text{|}r\left(g_{j}\right)=1,\;j<i\right\}\;if\;es_{max}>0\\ \left\{g_{j}\text{|}r\left(g_{j}\right)=1,\;j\geq i\right\}\;if\;es_{max}<0\;\\ \left\{\emptyset \right\}\;if\;es_{max}=0\; \end{array}$$

where i represents the position number of $\;es_{max}$ .

#### 2.3.4 Refining the Results From Leading-Edge Subsets

Some genes or gene sets with interactions appear more frequently than others, indicating their pivotal roles in the enriched KEGG pathways. For instance, the interacting gene set Raf1–Map2k1–Erk plays an important role in a series of pathways, such as the ErbB signaling, FoxO signaling, and Ras signaling pathways. The Apriori algorithm designed for finding frequent item sets was introduced to identify these frequently appearing genes or interacting gene sets (Agrawal and Srikant, 1994). The goal of the algorithm is to identify genes (including a single gene or multiple genes with interactions) whose frequency of occurrence in the gene interactions is greater than a specified threshold. Prior knowledge of gene–gene interactions was determined by the Reactome database (Croft et al., 2011) (https://reactome.org/download/tools/ReatomeFIs/FIsInGene_122220_with_annotations.txt). After the frequent gene sets were calculated by the Apriori algorithm, a new statistic d was defined to comprehensively evaluate the real weights of a frequent gene set. Finally, by sorting the modulus of the statistic d , the significant genes were obtained for further experiments.

The number of iterations was set to 4, which meant that gene interactions were generated with a maximum item size of 4. As Apriori uses a “bottom up” approach, genes in the union of the leading-edge subsets were used as the first-level candidates. Afterward, the number of genes in a candidate was increased by one, and the candidates that had an infrequent pattern (defined by the threshold) or were not consistent with the gene interaction knowledge were pruned. According to the above method, frequent candidates were extended one gene at a time. The threshold was set to 3, indicating that a candidate would be removed if its frequency was less than 3 among all transactions. The algorithm terminated when no frequent candidates could be generated, and frequent gene sets were identified by screening. Since the frequent gene sets were calculated based on prior annotation, a new statistic d was defined to comprehensively evaluate the real weights of a frequent gene set. The statistic d of a frequent gene set is equal to the multiplication between the column vector of the expression level and its correlation matrix, and the modulus of the statistic d represents comprehensive expression based on prior annotation and the actual expression level. Finally, by sorting the modulus of the statistic d , the significant genes were obtained for further experiments.

##### 2.3.4.1 Generating the Correlation Matrixes From a Gene Set

According to prior knowledge in the Reactome database, there are three categories of gene effects on another: upregulation, downregulation, or no effect, where the symbols ‘ → ’ and ‘⇢’ represent upregulation and downregulation, respectively. For a vector containing n genes, its correlation matrix M was a square matrix of order n , whose elements were the interaction information among these genes. We defined its element $a_{ij}$ as follows:

$$a_{ij}=\{\begin{array}{c} 1\;if\;g_{i}\to g_{j}\;\mathrm{and}\;i\neq j\\ -1\;if\;g_{i}\cdots >g_{j}\;\mathrm{and}\;i\neq j\\ 0\;if\;g_{i}\;\mathrm{had}\;\mathrm{no}\;\mathrm{effect}\;\mathrm{on}\;g_{j}\;\mathrm{and}\;i\neq j\\ 1\;if\;i=j \end{array}$$

where i and j represent the index numbers of $g_{i}$ and $g_{j}$ in the tuple, respectively.

For example, for a 4-dimensional vector of a gene set (A, B, C, D) with the following relationship:

{(A→B), (A⋯>C), (A→D), (B⋯>C), (C→D)} (1)

the correlation matrix was described as follows:

$$M=\left[\begin{array}{cccc} 1 & 1 & -1 & 1\\ 0 & 1 & -1 & 0\\ 0 & 0 & 1 & 1\\ 0 & 0 & 0 & 1 \end{array}\right]$$

##### 2.3.4.2 Generating Interactions From the Leading-Edge Subsets

Genes from the union of the leading-edge subsets were used to iteratively generate gene transactions. After obtaining the union of the leading-edge subsets with m genes, the vector l consisting of these genes and the correlation matrix M reflecting their interactions were created. Elements in l were considered basic gene interactions in the first iteration. For an iteration vector, a gene would be appended to a gene transaction if it had an interaction (upregulation or downregulation) with the gene at the end of the transaction, and all possible new interactions were used as the basic gene interactions for the next iteration. In the study, gene interactions with a maximum of 4 items were generated.

For example, let the 4-dimensional vector l=(A, B, C, D) be the union of the leading-edge subsets, and let the interactions be as described above in (1). For 4 iterations, the sequences of gene interactions $I=\left(I_{1},I_{2},I_{3},I_{4}\right)$ were generated as follows:

$$I_{1}=\left\{\begin{array}{c} \left(\text{A}\right)\\ \left(\text{B}\right)\\ \left(\text{C}\right)\\ \left(\text{D}\right) \end{array}\right\}\;\;I_{2}=\left\{\begin{array}{c} \left(\text{A},\text{ B}\right)\\ \left(\text{A},\text{ C}\right)\\ \left(\text{A},\text{ D}\right)\\ \left(\text{B},\text{ C}\right)\\ \left(\text{C},\text{D}\right) \end{array}\right\}\;I_{3}=\left\{\begin{array}{c} \left(\text{A},\text{ B},\text{ C}\right)\\ \left(\text{A},\text{ C},\text{ D}\right)\\ \left(\text{B},\text{ C},\text{ D}\right) \end{array}\right\}\;I_{4}=\left\{\begin{array}{c} \left(\text{A},\text{ B},\text{ C},\text{ D}\right) \end{array}\right\}$$ (3)

##### 2.3.4.3 Mining Frequent Gene Sets by the Apriori Algorithm

To detect frequent gene sets from the gene transactions, the value support was used to evaluate the frequency of a gene set. For the vector a of a gene set, the support of a was defined as the total count of a in all transactions.

For example, the support of (A, C) in interactions I in (3) was 2 ( (A, C) in $I_{2}$ and (A, C, D) in $I_{3}$ ).

Given a threshold min for support (a threshold min=3 was set in this study), gene interactions $I=\left(I_{1},I_{2}\ldots I_{t}\right)$ generated from t iterations and setting both the frequent gene set and the infrequent gene set, F and $\bar{F}$ , to ∅ initially, the algorithm in the study was described in two steps below:

Step 1 Start with i=1 . Traverse all interactions in I . Append the interactions whose support≥min to F and the interactions whose support<min to $\bar{F}$ , respectively. Based on the Apriori principle, if a transaction is found to be infrequent, then all its super interactions are also infrequent. Remove the transaction in $I_{i+1}$ (if it exists) when a transaction in $\bar{F}$ belongs to the transaction in $I_{i+1}$ .

Step 2 Repeat Step 1 until no further successful extensions are found. F is the returned result.

For example, for the gene interactions I described above in (3) and a given threshold min=3 , the process of the Apriori algorithm was as follows:

$$i=1:\;I_{1}=\left\{\begin{array}{c} \left(\text{A}\right)\\ \left(\text{B}\right)\\ \left(\text{C}\right)\\ \left(\text{D}\right) \end{array}\right\}\;\;support=\left\{\begin{array}{c} \left(\text{A}\right):7\\ \left(\text{B}\right):6\\ \left(\text{C}\right):8\\ \left(\text{D}\right):6 \end{array}\right\}\;F=\left\{\begin{array}{c} \left(\text{A}\right)\\ \left(\text{B}\right)\\ \left(\text{C}\right)\\ \left(\text{D}\right) \end{array}\right\}\;\bar{F}=\emptyset$$

In i=1 , no interactions in $I_{2}$ were removed.

$$i=2:\;I_{2}=\left\{\begin{array}{c} \left(\text{A},\text{ B}\right)\\ \left(\text{A},\text{ C}\right)\\ \left(\text{A},\text{ D}\right)\\ \left(\text{B},\text{ C}\right)\\ \left(\text{C},\text{D}\right) \end{array}\right\}\;\;support=\left\{\begin{array}{c} \left(\text{A},\text{ B}\right):3\\ \left(\text{A},\text{ C}\right):2\\ \left(\text{A},\text{ D}\right):1\\ \left(\text{B},\text{ C}\right):2\\ \left(\text{C},\text{D}\right):4 \end{array}\right\}\;F=\left\{\begin{array}{cc} \left\{\begin{array}{c} \left(\text{A}\right)\\ \left(\text{B}\right)\\ \left(\text{C}\right)\\ \left(\text{D}\right) \end{array}\right\} & \left\{\begin{array}{c} \left(\text{A},\text{B}\right)\\ \left(\text{C},\text{D}\right) \end{array}\right\} \end{array}\right\}\;\bar{F}=\left\{\begin{array}{c} \left(\text{A},\text{C}\right)\\ \left(\text{A},\text{D}\right)\\ \left(\text{B},\text{C}\right) \end{array}\right\}$$

In i=2 , (A, C, D) and (B, C, D) were super interactions in $\bar{F}$ and were removed in $I_{3}$ .

$$i=3:\;I_{3}=\left\{\begin{array}{c} \left(\text{A},\text{ B},\text{ C}\right) \end{array}\right\}\;\;support=\left\{\left(\text{A},\text{ B},\text{ C}\right):2\right\}\;F=\left\{\begin{array}{cc} \left\{\begin{array}{c} \left(\text{A}\right)\\ \left(\text{B}\right)\\ \left(\text{C}\right)\\ \left(\text{D}\right) \end{array}\right\} & \left\{\begin{array}{c} \left(\text{A},\text{B}\right)\\ \left(\text{C},\text{D}\right) \end{array}\right\} \end{array}\right\}\;$$

$$\;\bar{F}=\left\{\begin{array}{cc} \left\{\begin{array}{c} \left(\text{A},\text{C}\right)\\ \left(\text{A},\text{D}\right)\\ \left(\text{B},\text{C}\right) \end{array}\right\} & \left\{\begin{array}{c} \left(\text{A},\text{B},\text{C}\right) \end{array}\right\} \end{array}\right\}$$

In i=3 , (A,B, C, D) was a super transaction in $\bar{F}$ and was removed in $I_{4}$ . Iteration was terminated, and the frequent gene set F was obtained.

##### 2.3.4.4 Calculating the Weights of the Frequent Gene Set

After the frequent gene set was obtained by the Apriori algorithm, a new statistic d was introduced to evaluate the real ‘weights’ of the frequent gene set consisting of expression levels obtained from RNA-Seq. Given an element vector g from the frequent gene set, d was defined as the multiplication of its expression vector δ on its correlation matrix M :

d=δM

For example, given an element vector g=(A,B) from the frequent gene set F , its expression vector δ=(a,b) and correlation matrix $M=\left[\begin{array}{cc} 1 & 1\\ 0 & 1 \end{array}\right]$ [derived from the relationship (A → B)] were:

$$d=\left[\begin{array}{cc} a & a+b \end{array}\right]$$

The modulus of d should be:

$$\|d\|=\sqrt{a^{2}+{\left(a+b\right)}^{2}}$$

Since gene B was upregulated by gene A according to the prior knowledge, theoretically, if the expression value a of gene A was increased (a>0) , its expression value b should also be increased (b>0) . However, under the same condition where the expression value a was increased (a>0) , if the expression value b was decreased (b<0) , or if the increase in the expression value b was not as large as in the first case, ‖d‖ would be smaller in the second case than in the first case, indicating that the weights in the second case were smaller than those in the first case. Specifically, for a single gene  S , ‖d‖ is the absolute value of its expression level:

$$\|d\|=\|\left(s\right)\left[\begin{array}{c} 1 \end{array}\right]\|=\left|s\right|$$

Any frequent gene set could be described by d no matter how complicated the interactions among genes, and its ‖d‖ represented the comprehensive weights related to their interactions and expression levels.

For instance, given the 4-dimensional vector of gene set l=(A, B, C, D) , its expression vector δ=(a,b,c,d) and its correlation matrix M given above, its comprehensive weights were described as follows:

$$d=\left(a,b,c,d\right)\left[\begin{array}{cccc} 1 & 1 & -1 & 1\\ 0 & 1 & -1 & 0\\ 0 & 0 & 1 & 1\\ 0 & 0 & 0 & 1 \end{array}\right]=\left[\begin{array}{cccc} a & a+b & -a-b+c & a+c+d \end{array}\right]$$

$$\|d\|=\sqrt{a^{2}+{\left(a+b\right)}^{2}+{\left(-a-b+c\right)}^{2}+{\left(a+c+d\right)}^{2}}$$

According to this method, the weights associated with any frequent gene set will be referred to as its value of ‖d‖ , and the frequent gene set with the highest weights could then be identified by ranking the value of ‖d‖ . Since the value of ‖d‖ will increase with the increase in the number of items, the value of ‖d‖ must be compared among frequent gene sets with the same number of genes.

### 2.4 RT-qPCR Validation for the Candidate Genes

RT-qPCR was performed to validate the candidate genes. Total RNA from retinas was isolated and procedures for reverse transcription and Real-Time PCR were described previously earlier. Gene expression levels were quantified using the 2^−ΔΔCt^ method and normalized to GAPDH levels (Livak and Schmittgen, 2001). Graphical representation of the results was performed using GraphPad Prism v8.3.0 (GraphPad Software, Inc.).

## 3 Results

### 3.1 Data Preparation and Normalization

**FIGURE 1:**
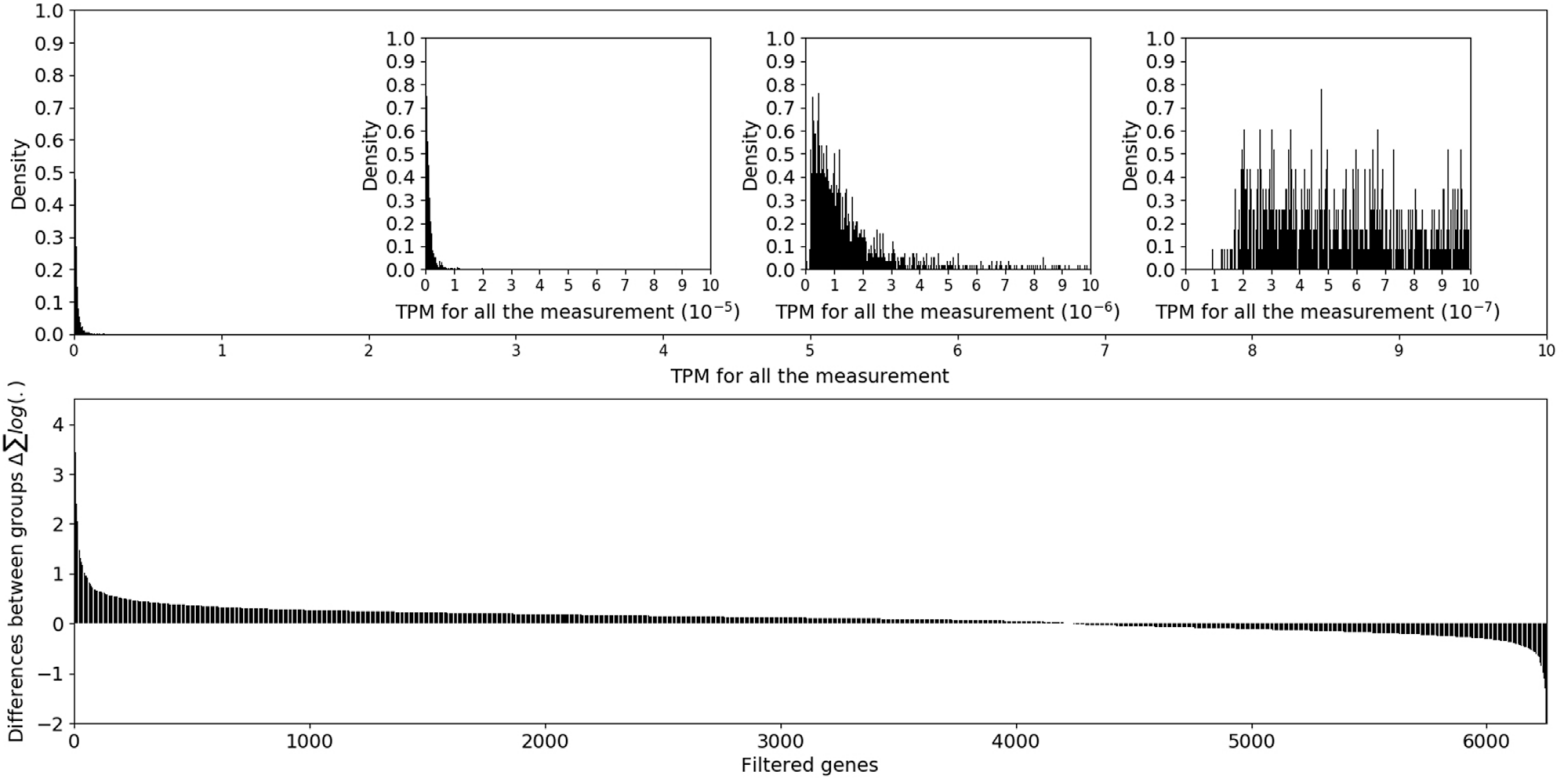
The upper panel showed the overall distribution of mRNA TPM values, and the lower panel showed the log2-transformed fold-change in filtered genes.

A total of 115,140 assembled transcript sequences were obtained from the clean data, among which 75,959 were distinct mRNA sequences. After TPM normalization, the expression levels of genes were observed on a 10-base logarithmic scale. The overall TPM values showed a one-tailed distribution, which was consistent with previous literature reports (Wagner et al., 2013; Bush et al., 2018; Monaco et al., 2019). Figure 1 shows that several genes had TPM values of 10^−4^, an amount of which was so small that it could not be clearly observed on the histogram. In addition, the TPM distribution on the order of 10^−7^ was irregular and inconsistent with the one-tailed distribution of the previous orders of magnitude. TPM data below 10^−6^ were excluded from further analysis. The expression differences of filtered genes were determined based on the log2-transformed fold-change TPM values. After miR-124-3p was knocked down in the mouse retina, most genes in the anti-miR-124 group were upregulated compared to the NC group, while a small portion were downregulated.

### 3.2 Identified Enriched KEGG Pathways and Their Leading-Edge Subsets

**FIGURE 2:**
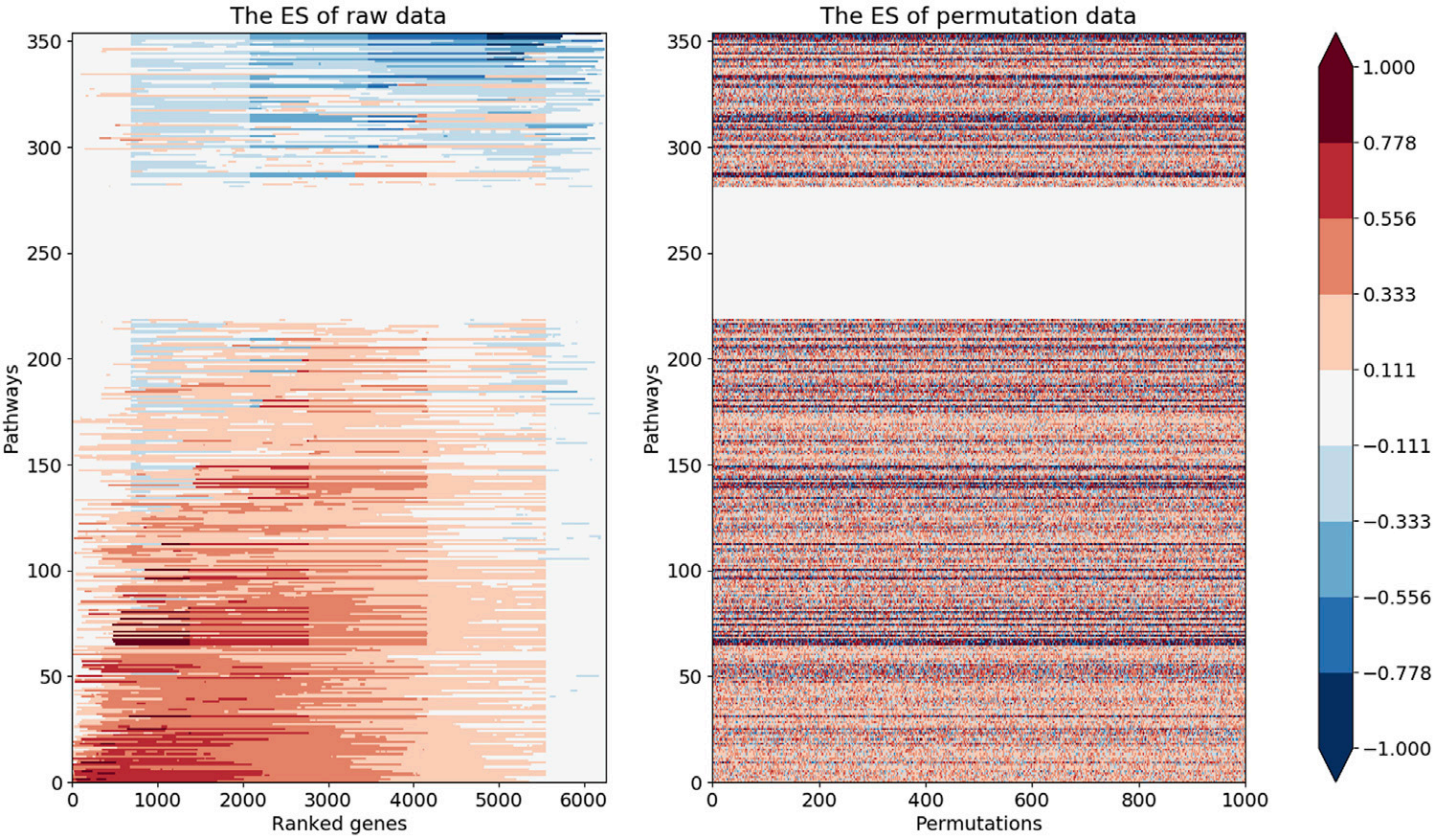
The left heatmap demonstrated ES values for all the 354 pathways in GSEA. The right heatmap illustrated randomly generated maximum ES values for the corresponding pathways.

**FIGURE 3:**
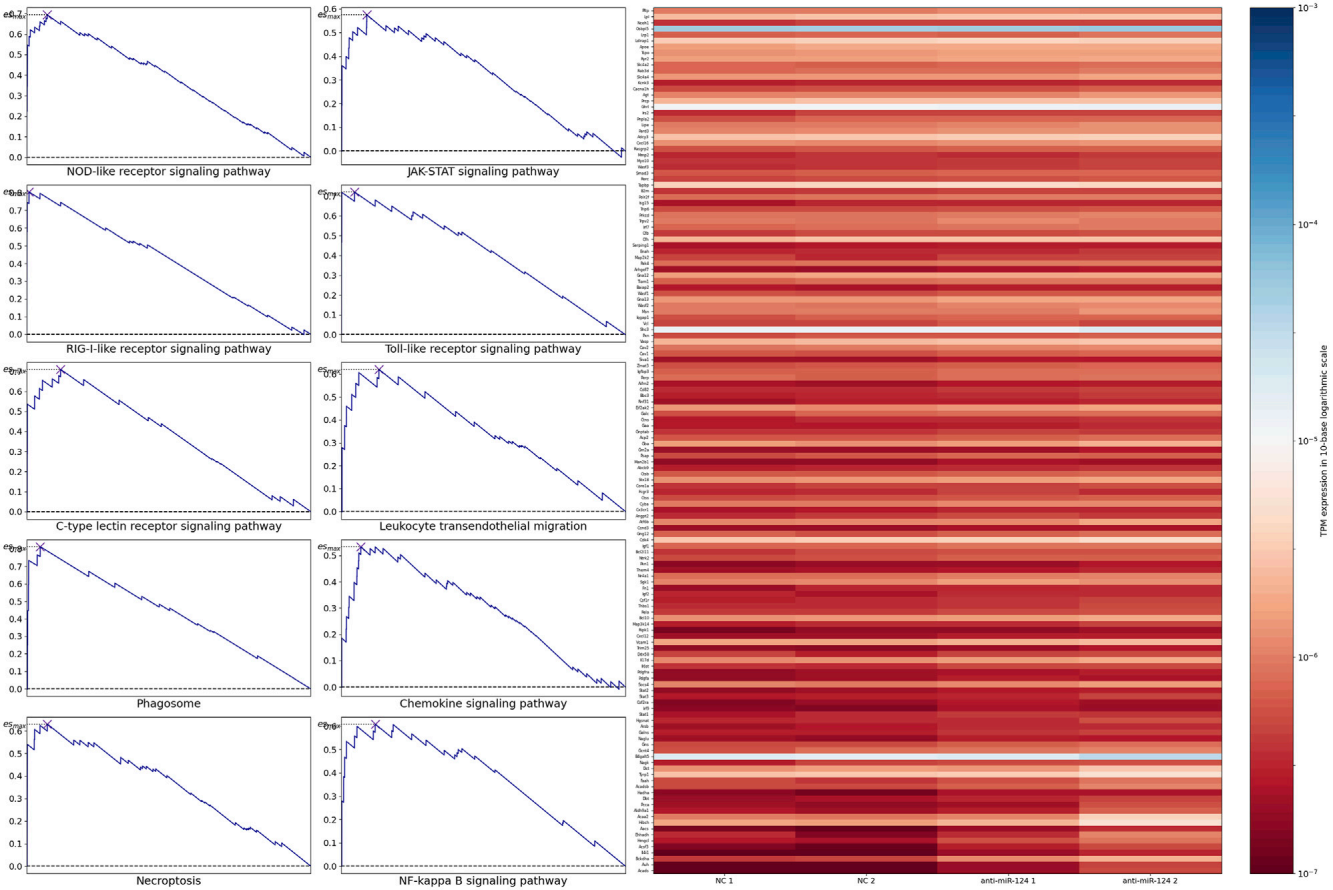
The left panel indicated the top 10 enriched pathways. The right panel indicated the expression level of the union of their leading-edge subsets.

Based on GSEA of 354 pathways, the ranked gene list was enriched at the top in most of the pathways and enriched at the bottom in a small number of pathways. After *p* values were calculated based on 1,000 permutations, pathways related to retinal development with *p* values <0.05 were selected (Figure 2). The results indicated that only pathways with a positive maximum ES showed significant enrichment in upregulated genes, consistent with the negative regulation of mRNA by miRNA. The top 10 enriched pathways and the expression levels of their leading-edge subsets are shown in Figure 3: the NOD-like receptor signaling pathway, C-type-like lectin receptor signaling pathway, phagosome, necroptosis, JAK-STAT signaling pathway, Toll-like receptor signaling pathway, leukocyte transendothelial migration, chemokine signaling pathway, NF-kappa B signaling pathway and RIG-I-like signaling pathway.

### 3.3 The Weights of Frequent Gene Sets From the Leading-Edge Subsets

**FIGURE 4:**
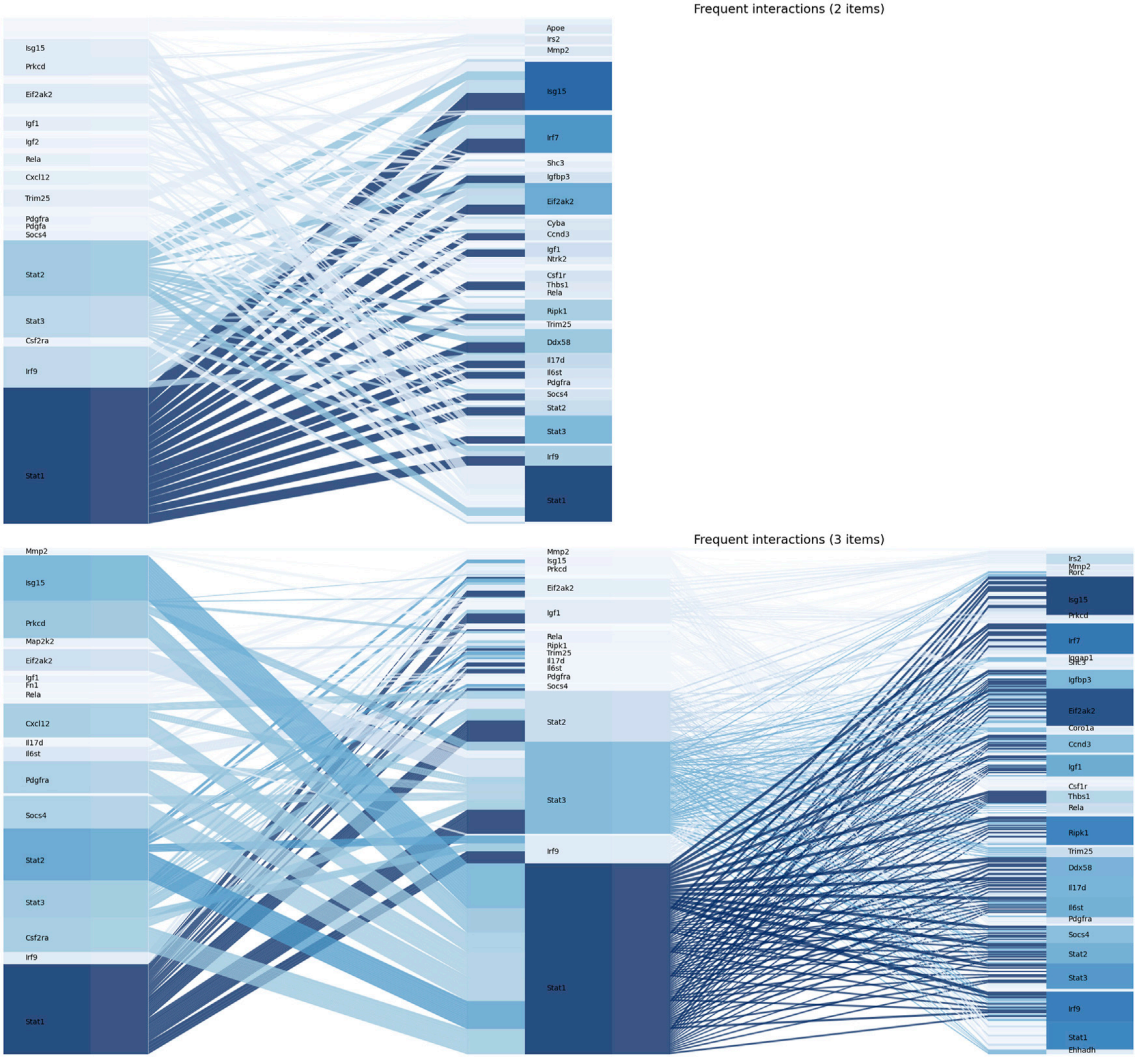
The figure illustrated d values and their interactions in frequent gene sets. The upper panel showed the expression patterns of frequent gene sets with two items, while the lower panel showed the expression patterns of frequent gene sets with 3 items.

**FIGURE 5:**
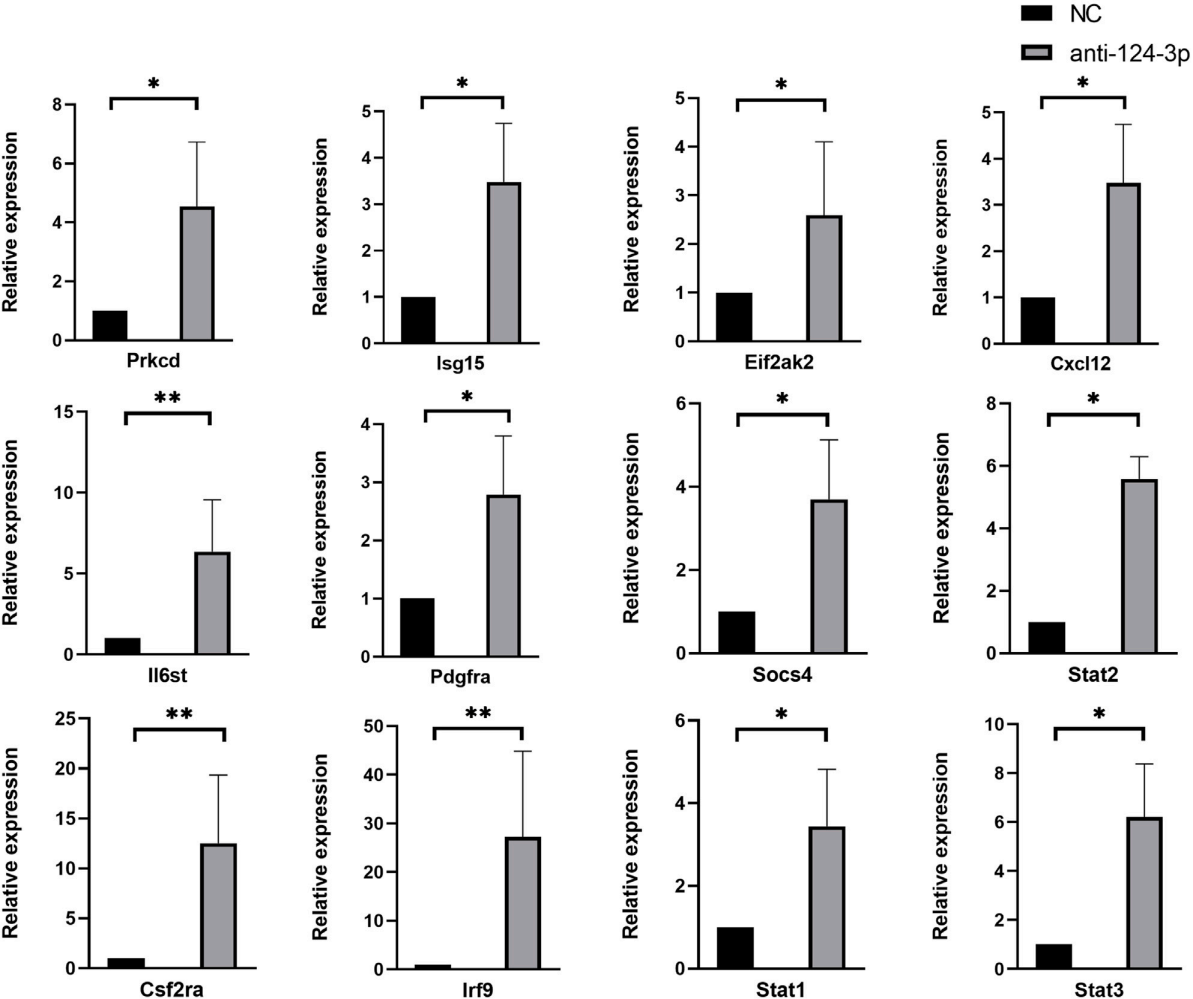
Results of RT-qPCR showed the expression of candidate genes bewteen NC and anti-124 group, which exhibited a similar trend to the RNA-Seq results. Each RT-qPCR experiment was repeated three times with independently isolated RNA samples. Results were presented as mean ± standard deviation (anti-miR-124 group, miR-124-3p knockdown group; NC group, negative control group; *p < 0.05, **p < 0.01)

The Apriori algorithm was performed on the union of the leading-edge subsets to identify the frequent gene set. After the first iteration, 60 single genes were identified as frequent. Based on this, frequent gene sets with 2 and 3 genes were further identified, and no frequent gene sets were found with more than 4 genes. Values of ‖d‖ for frequent gene sets with 2 and 3 genes were calculated. In Figure 4, each colored line represents an interaction. The wider the height of the color line is, the greater the value of ‖d‖ . The background color of the gene represents the interactions between the gene and its downstream genes. The darker the background color of the gene is, the higher the number of interactions. Upstream of the frequent interactions with two genes, Prkcd, Igf1, Irf9, Cxcl12, Trim25, Stat2, Stat3 and Stat1 had a considerable ‖d‖ value and interactions with the downstream genes. For frequent interactions with 3 genes in the first set, genes with a considerable ‖d‖ value and interactions were Isg15, Prkcd, Eif2ak2, Cxcl12, Il6st, Pdgfra, Socs4, Stat2, Stat3, Csf2ra, Irf9 and Stat1. In summary, Prkcd, Irf9, Stat3, Cxcl12, Stat1 and Stat2 had the greatest interactions and the greatest value of ‖d‖ to downstream genes among all frequent transactions, indicating that they played a pivotal role in the downstream genes of miR-124-3p. The frequent gene sets and their weights are shown in Data Sheet 2. RT-qPCR was performed to validate the expression of candidate genes. These genes exhibited a similar trend to the RNA-Seq results (Figure 5). Primer sequences were described in Data Sheet 3.

## 4 Discussion

After gain and loss intervention of the upstream miRNA in a functional experiment, transcriptomics technologies such as RNA-Seq make it possible to obtain a large amount of downstream gene expression data in a single experiment. To handle high-throughput data, although there are many other methods based on the entire gene set analysis, such as CePa (Gu et al., 2012) and SPIA (Tarca et al., 2009), GSEA is still one of the most widely used and outperformed methods by focusing on the entire gene set rather than a single threshold (Bayerlová et al., 2015). Using GSEA, we analyzed the ranked gene list after knockdown of miR-124-3p to determine if they were enriched in given phenotypes, that is, KEGG pathways related to retinal development in our study. In the results, most genes in the anti-miR-124 group were upregulated compared to the NC group, and only pathways in upregulated genes showed significant enrichment, which was consistent with prior theories. A series of KEGG pathways with significant enrichment could be obtained as well as their leading-edge subsets of their associated genes by GSEA. However, for a low-throughput functional experiment, only one or several downstream genes and pathways were selected. The number of generated candidate genes is usually still too high and needs further refinement.

Since not all genes play an equally important role in a pathway, we hypothesized that the genes that have the most interactions with other genes in the entire leading-edge subsets are likely to be the most important. To obtain those genes or gene sets, interactions based on KEGG pathway topology were generated, and the Apriori algorithm was performed. The Apriori algorithm is designed for mining the frequent item set. Although it has exponential time complexity, interactions were generated based on the correlation matrix of a , and the nonexistent gene interactions (with an element equal to 0) were excluded, which reduced the amount of computation from bottom to top.

This greatly decreased the calculation time of the Apriori algorithm, making the calculation time acceptable. Moreover, we introduced a new statistic d to evaluate the connection between the prior knowledge and its actual expression. The larger the value of d in a gene or gene set, the better the agreement between the KEGG pathway topology and its actual expression. Accordingly, we further refined the result of leading-edge subsets.

The statistic d was a corrected expression vector, considering the relationship and gene expressions. For genes having interactions, if the expression levels did not conform to the prior interactions among them, the value of ‖d‖ might still be small even if the expression levels of these genes were large. This made the modulus of ‖d‖ a good representative of the comprehensive value of the gene set. The comparison of relative biological importance among gene sets could be achieved by comparing their ‖d‖ values. By sorting the value of ‖d‖ , critical genes or gene sets were identified.

## 5 Conclusion

**FIGURE 6:**
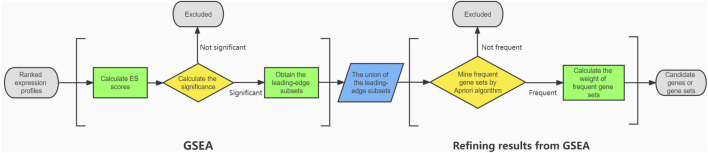
The flowchart of the proposed method.

In this study, through mRNA sequencing after miR-124-3p knockdown, GSEA was performed to identify significant KEGG pathways and their leading-edge subsets. We demonstrated that using the Apriori algorithm and defining the statistic d was a novel way to refine the GSEA results (Figure 6). The actual expression level and its correlation of a gene set was combined and represented by a single number, the modulus of d . According to this information, a series of key downstream genes and gene sets were identified.

## Data Availability

The datasets presented in this study can be found in online repositories. The names of the repository/repositories and accession number(s) can be found in the article/Supplementary Material.

## References

[B1] Ackermann M. Strimmer K. (2009). A General Modular Framework for Gene Set Enrichment Analysis. BMC Bioinforma. 10, 47. 10.1186/1471-2105-10-47 PMC2661051 19192285

[B2] Agrawal R. Srikant R. (1994). “Fast Algorithms for Mining Association Rules,” in Proceedings of the 20th International Conference on Very Large Data Bases (United States: Morgan Kaufmann Publishers Inc.), 487–499.

[B3] Bayerlová M. Jung K. Kramer F. Klemm F. Bleckmann A. Beißbarth T. (2015). Comparative Study on Gene Set and Pathway Topology-Based Enrichment Methods. BMC Bioinforma. 16, 334. 10.1186/s12859-015-0751-5 PMC4618947 26489510

[B4] Bush S. J. Freem L. MacCallum A. J. O’Dell J. Wu C. Afrasiabi C. (2018). Combination of Novel and Public RNA-Seq Datasets to Generate an mRNA Expression Atlas for the Domestic Chicken. BMC genomics 19, 594. 10.1186/s12864-018-4972-7 30086717 PMC6081845

[B5] Croft D. O'Kelly G. Wu G. Haw R. Gillespie M. Matthews L. (2011). Reactome: a Database of Reactions, Pathways and Biological Processes. Nucleic acids Res. 39, D691–D697. 10.1093/nar/gkq1018 21067998 PMC3013646

[B6] Damiani D. Alexander J. J. O'Rourke J. R. McManus M. Jadhav A. P. Cepko C. L. (2008). Dicer Inactivation Leads to Progressive Functional and Structural Degeneration of the Mouse Retina. J. Neurosci. 28, 4878–4887. 10.1523/JNEUROSCI.0828-08.2008 18463241 PMC3325486

[B7] Dillies M.-A. Rau A. Aubert J. Hennequet-Antier C. Jeanmougin M. Servant N. (2013). A Comprehensive Evaluation of Normalization Methods for Illumina High-Throughput RNA Sequencing Data Analysis. Briefings Bioinforma. 14, 671–683. 10.1093/bib/bbs046 22988256

[B8] Gu Z. Liu J. Cao K. Zhang J. Wang J. (2012). Centrality-based Pathway Enrichment: a Systematic Approach for Finding Significant Pathways Dominated by Key Genes. BMC Syst. Biol. 6, 56. 10.1186/1752-0509-6-56 22672776 PMC3443660

[B9] Ha M. Kim V. N. (2014). Regulation of microRNA Biogenesis. Nat. Rev. Mol. Cell Biol. 15, 509–524. 10.1038/nrm3838 25027649

[B10] Hackler L. Wan J. Swaroop A. Qian J. Zack D. J. (2010). MicroRNA Profile of the Developing Mouse Retina. Invest. Ophthalmol. Vis. Sci. 51, 1823. 10.1167/iovs.09-4657 19933188 PMC2868396

[B11] Karali M. Peluso I. Gennarino V. A. Bilio M. Verde R. Lago G. (2010). miRNeye: a microRNA Expression Atlas of the Mouse Eye. BMC genomics 11, 715. 10.1186/1471-2164-11-715 21171988 PMC3018480

[B12] Karali M. Peluso I. Marigo V. Banfi S. (2007). Identification and Characterization of microRNAs Expressed in the Mouse Eye. Invest. Ophthalmol. Vis. Sci. 48, 509–515. 10.1167/iovs.06-0866 17251443

[B13] Karali M. Persico M. Mutarelli M. Carissimo A. Pizzo M. Singh Marwah V. (2016). High-resolution Analysis of the Human Retina miRNome Reveals isomiR Variations and Novel microRNAs. Nucleic Acids Res. 44, 1525–1540. 10.1093/nar/gkw039 26819412 PMC4770244

[B14] Livak K. J. Schmittgen T. D. (2001). Analysis of Relative Gene Expression Data Using Real-Time Quantitative PCR and the 2−ΔΔCT Method. Methods 25, 402–408. 10.1006/meth.2001.1262 11846609

[B15] Maleki F. Ovens K. Hogan D. J. Kusalik A. J. (2020). Gene Set Analysis: Challenges, Opportunities, and Future Research. Front. Genet. 11. 10.3389/fgene.2020.00654 PMC7339292 32695141

[B16] Monaco G. Lee B. Xu W. Mustafah S. Hwang Y. Y. Carré C. (2019). RNA-seq Signatures Normalized by mRNA Abundance Allow Absolute Deconvolution of Human Immune Cell Types. Cell Rep. 26, 1627–1640. e1627. 10.1016/j.celrep.2019.01.041 30726743 PMC6367568

[B17] Ritchie W. Rajasekhar M. Flamant S. Rasko J. E. J. (2009). Conserved Expression Patterns Predict microRNA Targets. PLoS Comput. Biol. 5, e1000513. 10.1371/journal.pcbi.1000513 19779543 PMC2736581

[B18] Subramanian A. Tamayo P. Mootha V. K. Mukherjee S. Ebert B. L. Gillette M. A. (2005). Gene Set Enrichment Analysis: A Knowledge-Based Approach for Interpreting Genome-wide Expression Profiles. Proc. Natl. Acad. Sci. U.S.A. 102, 15545–15550. 10.1073/pnas.0506580102 16199517 PMC1239896

[B19] Tarca A. L. Draghici S. Khatri P. Hassan S. S. Mittal P. Kim J.-s. (2009). A Novel Signaling Pathway Impact Analysis. Bioinforma. Oxf. Engl. 25, 75–82. 10.1093/bioinformatics/btn577 PMC2732297 18990722

[B20] Wagner G. P. Kin K. Lynch V. J. (2013). A Model Based Criterion for Gene Expression Calls Using RNA-Seq Data. Theory Biosci. 132, 159–164. 10.1007/s12064-013-0178-3 23615947

[B21] Wagner G. P. Kin K. Lynch V. J. (2012). Measurement of mRNA Abundance Using RNA-Seq Data: RPKM Measure Is Inconsistent Among Samples. Theory Biosci. 131, 281–285. 10.1007/s12064-012-0162-3 22872506

[B22] Wang Y.-P. Li K.-B. (2009). Correlation of Expression Profiles between microRNAs and mRNA Targets Using NCI-60 Data. BMC genomics 10, 218. 10.1186/1471-2164-10-218 19435500 PMC2686738

[B23] Wang Y. Wang X. Jiang Y. Liu R. Cao D. Pan J. (2020). Identification of Key miRNAs and Genes for Mouse Retinal Development Using a Linear Model. Mol. Med. Rep. 22, 494–506. 10.3892/mmr.2020.11082 32319662 PMC7248464

[B24] Wang Z. Gerstein M. Snyder M. (2009). RNA-seq: a Revolutionary Tool for Transcriptomics. Nat. Rev. Genet. 10, 57–63. 10.1038/nrg2484 19015660 PMC2949280

